# Association between Basal Metabolic Rate and Handgrip Strength in Older Koreans

**DOI:** 10.3390/ijerph16224377

**Published:** 2019-11-09

**Authors:** Sung-Kwan Oh, Da-Hye Son, Yu-Jin Kwon, Hye Sun Lee, Ji-Won Lee

**Affiliations:** 1 Department of Family Medicine, Yonsei University College of Medicine, 50 Yonsei-ro Seodaemun-gu, Seoul 03722, Korea; aryary@hanmail.net (S.-K.O.); sonda@yuhs.ac (D.-H.S.); 2 Department of Family Medicine, Yong-In Severance Hospital, 23 Yongmunno (405 Yeokbuk-dong), Gyeonggi 17046, Korea; digda3@yuhs.ac; 3 Biostatistics Collaboration Unit, Department of Research Affairs, Yonsei University College of Medicine, 50-1 Yonsei-ro, Seodaemoon-gu, Seoul 03722, Korea; hslee1@yuhs.ac

**Keywords:** hand strength, muscle strength dynamometer, basal metabolic rate, sarcopenia, Koreans

## Abstract

We investigated the relationship between the basal metabolic rate (BMR) and muscle strength through measurement of handgrip strength. We conducted a cross-sectional study of a population representative of older Korean from the 2014–2016 Korean National Health and Nutrition Examination Survey. A total of 2512 community-dwelling men and women aged 65 years and older were included. The BMR was calculated with the Singapore equation and handgrip strength was measured using a digital dynamometer. The patients were categorized into handgrip strength quartiles and a weighted one-way analysis of variance (ANOVA) for continuous variables and a weighted chi-squared test for categorical variables were performed. Pearson, Spearman correlation analysis, univariate, and multivariate linear regression were performed. Analysis of covariance (ANCOVA) was also performed to determine the association between basal metabolic rate and handgrip strength quartiles after adjusting for confounding factors. The BMR increased according to handgrip strength quartile after adjusting for age, BMI, relative fat mass, comorbidity number, resistance exercise, aerobic physical activity, household income, educational level, smoking status, and alcohol ingestion in both sexes (*p* < 0.001). Handgrip strength has a positive association with the BMR in older Korean people. Therefore, muscle strength exercises should be considered for regulating the BMR in the older people.

## 1. Introduction

The basal metabolic rate (BMR) is defined as the energy required performing essential physical functions at rest [1]. Although an abnormally high metabolic rate is associated with some pathologic conditions or inflammatory conditions [2], BMR tends to decrease with advancing age [1], and a low BMR plays an important role in the pathogenesis of the obesity and age related chronic disease in old age [2]. 

Energy metabolism and body composition are closely related to fat-free mass (FFM). Although the FFM is known as a primary determinant of BMR [1], it can only account for between 50% and 70% of the BMR [3]. Apart from the FFM, several other factors such as heritable, physiological, and genetic factors could be also considered determinants of BMR [4]. 

Sarcopenia has been used to define the age-related loss of skeletal muscle mass and strength, which are associated with a poor quality of life and loss of independence in older people [5,6,7]. Muscle mass aside, loss of muscle strength in the elderly has been shown to increase risk of poor physical performance. Additionally, previous studies reported that older people with decreased muscle strength showed higher risk of falls, frailty, and mortality, independent of muscle mass [5,6,7]. 

Studies have demonstrated decreased BMR to be associated with sarcopenia or with loss of muscle mass [8,9,10]. However, little is known about the relationship between BMR and muscle strength. Muscle strength and muscle mass do not necessarily correlate with or affect each other, a finding that again stresses the importance of muscle performance in older people [11].

In recent study, short-term resistance training in 19 apparently healthy women lead to a significant increase in BMR (*p* < 0.001) without any changes in body composition, including body fat, FFM, and body mass index (BMI) [12], which indicates that change in body composition is not the sole mechanisms for change in BMR. In this context, further study is needed to demonstrate association between BMR and muscle strength.

Muscle strength is measured using hand-grip equipment (isometric strength, isokinetic power, etc.) [13]. Hand-grip strength (HGS) is assessed by simple, fast, and standardized measurements of overall muscular strength [6,14]. Measurement of HGS also makes it possible to predict disability and frailty in the older people [15]. 

Until now, there have been no studies on the association between BMR and muscle strength alone. Our nationwide population-based study aims to determine the relationship between BMR and HGS in elderly Koreans

## 2. Materials and Methods 

### 2.1. Survey Overview and Study Population

**Figure 1:**
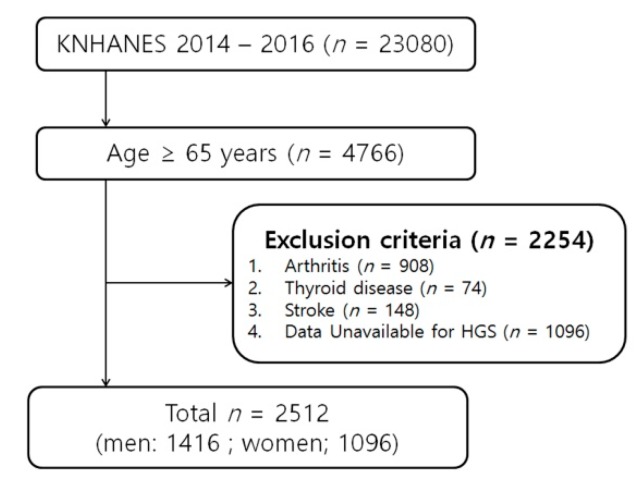
Study population flowchart diagram. KNHANES, Korea National Health and Nutrition Examination Survey.

This cross-sectional study was conducted using data from the Korean National Health and Nutrition Examination Survey (KNHANES) provided by the Korea Centers for Disease Control and Prevention (KCDC) for 2014–2016. KNHANES is a nationwide cross-sectional survey that assesses the health and nutritional status of Koreans. KNHANES reports and microdata are released annually and are available to the public free of charge at the end of the following year. KCDC also published documents on survey manuals through the official website of KNAHNES (http://knhanes.cdc.go.kr) [16]. Sampling was performed using a stratified, multi-staged, probability-sampling design based on the age, sex, and geographical area of the participants via household registries. In this study, data from 4766 individuals aged 65 years and older were included from the 2014–2016 KNHANES (*n* = 23,080). Of these individuals, we excluded those who met the following criteria (*n* = 2254): presence of osteoarthritis, rheumatoid arthritis; history of stroke, or thyroid disease; and those whose data were unavailable to evaluate HGS. After excluding these individuals, 2512 participants were included in the final analysis (Figure 1). The average age of this study population was 72.6 years, and the median age was 72 years. The oldest individual was 80 years.

### 2.2. Data Collection

The 2014–2016 KNHANES included demographic, health, social, and nutritional data collected via a three-component survey method. Information regarding age, household income, and residence was collected through a health interview, whereas information on health-related behaviors, such as participation in resistance exercise, aerobic physical activity, smoking habits, and drinking status was obtained from self-report questionnaires. The standardized questionnaire was developed by KCDC and questionnaire was reviewed and validated annually by health indicators standardization subcommittee of KCDC. Health examinations included body measurements (height, weight, and waist circumference), blood pressure, and laboratory tests. Smoking status was assessed according to participants’ answers to the question “Do you currently smoke?” Participants were considered to be current smokers if they answered “I smoke every day” or “I sometimes smoke”, and reported that they had smoked more than five packs (100 cigarettes) in their whole life. Participants were asked about average amount and frequency of alcoholic consumption for the month preceding the interview. Alcohol use was defined as drinking more than two to three days per week. Physical activity was assessed by asking participants how often they engaged in exercise each week using a Korean version of the international physical activity questionnaire. Aerobic physical activity was defined as moderate-intensity activity greater than or equal to 2.5 h per week or a combination of moderate- and high-intensity activity greater than or equal to 1.25 h per week [17]. Frequency of resistance exercise was assessed according to participants’ answers to the question “How many times do you do resistance exercise (push-ups, sit-ups, lifting dumbbells or barbells) a week?” The resistance exercise group included participants who performed resistance exercise greater than or equal to three times per week [17]. Height and weight were recorded to the closest 0.1 cm (Seca 225; Seca GmbH, Hamburg, Germany) and 0.1 kg (GL-6000-20; G-tech, Seoul, Korea), respectively. Body mass index (BMI) was calculated as the weight in kilograms divided by the square of the height in meters (kg/m^2^). HGS was estimated using a digital hand dynamometer (Digital Grip Strength Dynamometer, T.K.K 5401; Takei Scientific Instruments Co., Ltd., Tokyo, Japan). HGS was measured using a standard grip test as specified by the American Society of Hand Therapy, in a standing position with arms to the side and elbows fully extended at the thigh level [18]. Participants were asked to apply the maximum grip strength using both the left and right hands, three times for each hand. A break interval of at least 30 seconds between each measurement was allowed [19]. HGS was defined as the maximum grip strength of the dominant hand [20].

### 2.3. Definition of Comorbidity Number

We constructed a simple comorbidity index (range 0–13), where one point was added for each comorbidity (hypertension, diabetes mellitus, dyslipidemia, myocardial infarction, angina, chronic renal failure, hepatitis, liver cirrhosis, malignancy, asthma, pulmonary tuberculosis, atopic dermatitis, and depression). A previous study applied a similar approach [21].

### 2.4. Definitions of BMR

The BMR was calculated by the Singapore equation as indicated below [22]. 

For men:

BMR (kJ/d) = 52.6 × weight (kg) + 2788(1)

For women:

BMR (kJ/d) = 52.6 × weight (kg) + 1960(2)

A previous study proved that Singapore equation was the most accurate tool to predict BMR in Chinese population. BMR is used interchangeably with the resting metabolic rate (RMR) due to their similar measurements and definitions in this study [23].

### 2.5. Definitions of Fat Mass

The 2014–2016 KNHANES does not contain direct measurements of fat mass and FFM, so we calculated the fat mass using relative fat mass (RFM) index, which was introduced by Orison et al. as shown below [24]. These authors obtained the RFM index using American National Health and Nutrition Examination Survey (NHANES) 1999–2004 data, demonstrating that this index was more accurate than BMI to estimate whole-body fat percentage in women and men [23]. RFM index was validated in the Korean population in a previous study [25].

For men,

RFM = 64 − (20 × ((height (m)) / waist (m)))(3)

For women, 

RFM = 76 − (20 × ((height (m)) / waist (m))).(4)

### 2.6. Statistical Analysis

Sample weighting and complex sampling were used to obtain a representative sample of the older Korean population. Both female and male participants older than or equal to 65 years of age were classified into quartiles based on HGS ((men: Q1, ≤29.1 kg; Q2, 29.2–33.9 kg; Q3, 34.0–38.1 kg; and Q4, 38.2–59.4 kg) (women: Q1, ≤16.8kg; Q2, 16.9–20.5 kg; Q3, 20.6–23.8 kg; and Q4, 23.9–37.1 kg)). The results are expressed as the mean and standard deviation (SD) or number (percentage) for quantitative variables. The analysis of subject characteristics according to HGS quartiles was performed using a weighted one-way analysis of variance for continuous variables and a weighted chi-squared test for categorical variables. Pearson’s correlation and Spearman correlation, and univariate and multivariate linear regression were performed. Multicollinearity was evaluated by estimating the variance inflation factor (VIF). The conventional criterion for absence of multicollinearity (VIF < 10) was used. An analysis of covariance (ANCOVA) was performed using a general linear model approach to determine the association between the BMR and HGS quartiles after adjusting for confounding factors such as age, BMI, RFM, resting exercise, aerobic physical activity, comorbidity number, household income, educational level, smoking status, and alcohol use. Statistical analyses were performed with SPSS software (version 23.0, SPSS Inc., Chicago, IL, USA). *p*-Values less than 0.05 were considered statistically significant.

### 2.7. Ethics Statement

The study protocol was reviewed and approved by the institutional review board of the Korea center for Disease Control and Prevention (approval no. 2013-12EXP-03-5C, 2015-01-02-6C). Informed consent was obtained from all participants when the 2014–2016 KNHANES was conducted in accordance with the ethical principles of the Declaration of Helsinki.

## 3. Results

### 3.1. Clinical Characteristics of the Participants

The clinical characteristics of the participants are shown in Table 1. The total number of participants s included in the study were 1416 men (mean age = 72.1 ± 0.09 years) and 1096 women (mean age = 73.5 ± 0.1 years). The value of RFM was higher in women. The percentage of aerobic physical activity and resistance exercise was higher in men compared to women. The BMR and HGS were 6173.8 ± 8.6 kJ/day and 33.6±0.1 kg in men and 4875.08 ± 10.0 kJ/day and 20.2 ± 0.1 kg in women, respectively. 

Table 2 shows the demographic and clinical characteristics of participants according to HGS quartile. As HGS increased, the mean age tended to decrease. The mean BMI gradually increased in accordance with the HGS quartile (*p* < 0.001) for both sexes. Moreover, participants in the fourth quartile (strongest) of HGS had the highest socioeconomic position according to household income and education level. 

### 3.2. Association between BMR and Studied Variables

**Figure 2:**
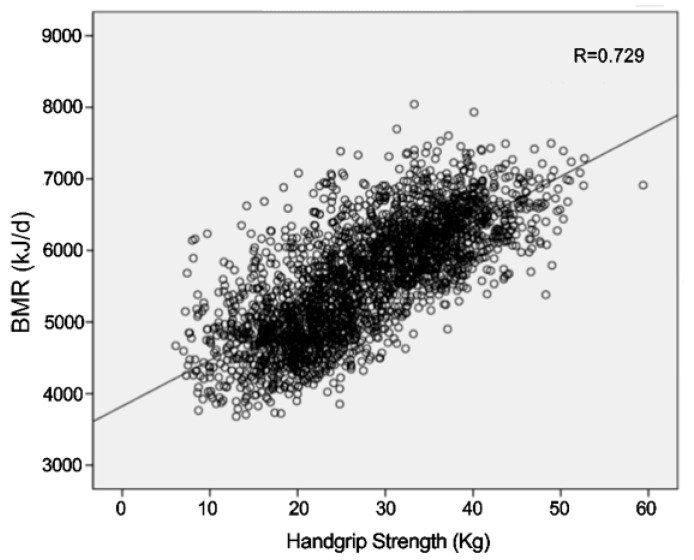
Plots of linear regression between basal metabolic rate and handgrip strength. The linear regressions are BMR (kJ/d) = 3824 + 64.2 × HGS (*r* = 0.729).

A Pearson correlation and Spearman correlation coefficient between BMR and studied variables in both men and women are presented in Table 3 and Table 4. HGS demonstrated significant correlations with BMR with coefficient value (*r* = 0.396, *p* < 0.001 in men, *r* = 0.333, *p* < 0.001 in women). In addition, HGS was significantly correlated with BMR in total population (*r* = 0.729, *p* < 0.001) (Figure 2). Multivariate analyses confirmed the independent associations between BMR and BMI, RFM, HGS, education level, comorbidity number in both men and women. 

### 3.3. Association between BMR and HGS

The BMR also significantly increased along with the HGS quartile in both men and women after adjusting for confounding factors (Table 5). 

## 4. Discussion

In this study, muscle strength by measured HGS was independently and positively associated with BMR in a dose-dependent manner after adjusting for confounding factors in both sexes. We used HGS for assess of muscle strength. HGS is a simple bedside measure that has emerged as an alternative assessment for muscle strength [6] and previous studies demonstrated that HGS can be used to early detection of age related disease such as impaired pulmonary function or cardiovascular diseases [17,26]. 

BMR decreases with the aging process [27] and is clinically important in old people. BMR can be used not only as a predictor of long-term weight gain [27] and the development of age-related chronic disease, but also as an object marker for frailty in older men [8]. Body composition also changes in many ways during the aging process, and sarcopenia has been used to define the age-related loss of both skeletal muscle mass and strength in older people [28]. Sarcopenia is associated with various chronic diseases, such as type 2 diabetes mellitus, chronic obstructive pulmonary disease, chronic heart failure, chronic kidney disease, and cancer [29].

Recent longitudinal studies showed a disassociation between muscle mass and muscle strength [11]. In healthy older women, physical performance has been correlated with strength in the lower limb but not with appendicular lean body mass [30]. Additionally, providing androgen or growth factor supplementation has resulted in a significant increase in only muscle mass but not in muscle strength or performance [31]. These findings suggest that muscle strength may play a different role in body muscle regardless of muscle mass. Indeed, the term “dynapenia” has been introduced, which is defined as an age-related loss of muscle strength and power that is not caused by muscular or neurologic diseases in older people [11]. In line with this definition of dynapenia, many studies have shown that decreased muscle strength contributes to decreased mobility and performance, frailty, and mortality, regardless of extent of muscle mass, in older population [5,6,7]. In this regard, when describing BMR, it may be necessary to distinguish between muscle mass and muscle strength. However, few studies have examined the relationship between the BMR and muscle strength. A previous study showed that heavy resistance-strength exercises increased RMR in healthy older people, nevertheless, this showed the limited mechanism only considering increased muscle mass [32]. Furthermore, most studies showing the efficacy of exercise and post-exercise physiology do not distinguish between muscle mass and strength.

A strength of our study is that this is the first study to describe a relationship between the BMR and the muscle strength itself in old population, which strongly suggest that increasing muscle strength besides muscle mass should be considered to improve their BMR in order people. 

Although the associations between BMR and muscle strength are not fully understood, several possible mechanisms has been suggested. Muscle strength training is known to increase number of capillaries and mitochondria. Metabolic stressors induced by muscle strength training have the ability to stimulate mitochondrial biogenesis [33]. A metabolism is series of process that produce energy by oxidation reactions in mitochondria [34] and are usually examined by indirect calorimetry, which quantifies O2 consumption [34]. Also, increased number of capillaries leads to increase in metabolic rate through increasing oxygen exchange capacity [35]. Next, muscle strength training also increases growth hormone (GH) [36], which is associated with the RMR, regardless of changes in body composition [37]. Further prospective and experimental studies are needed to verify the direct associations between BMR and muscle strength in old ages.

This study has several limitations. First, this study was cross-sectional, limiting our ability to conclude causation. Second, it is also possible that we did not exclude all the confounding factors that affect BMR and muscle strength. We could not directly measure and adjust thyroid hormone levels, which influence the BMR. However, we excluded people with osteoarthritis or rheumatoid arthritis, and thyroid disease and adjusted confounding variables of chronic diseases that could affect BMR and HSG. Third, muscle mass could not be measured, which is known as an independent determinant of the BMR [1], so the disassociation between muscle strength and muscle mass cannot be completely confirmed. Also, fat free mass was not included in our data so we calculated and adjusted RFM instead of FFM. Finally, we used a calculated BMR instead of an indirect measure using calorimetry, which estimates metabolic rate by measuring oxygen consumption and carbon dioxide production [38]. However, previous studies have demonstrated the reliability of a calculated BMR, and most estimates of BMR obtained for weight reduction interventions have relied on such calculated values [1]. Regardless of these limitations, this study provides a direction for further studies regarding optimal muscle strength exercises and BMR in older adults. 

## 5. Conclusions

In this cross-sectional study, we found an independent relationship between BMR and HGS. In the elderly, BMR significantly increased along HGS quartiles in both men and women after adjusting for confounding variables. Our results suggest that muscle strength itself may play an important role in regulating BMR, and that muscle strength exercises should therefore be considered when regulating BMR in older populations. Further studies are needed to clarify if muscle strength plays any causal role in BMR after adjusting for muscle mass and fat mass.

## Figures and Tables

**Table 1:** Clinical characteristics of study population.

Variable	Men (*n* = 1416)	Women (*n* = 1096)	*p*-Value *
Age (years)	72.1 ± 0.09	73.5 ± 0.1	<0.001
BMI (kg/m^2^)	23.6 ± 0.06	24.1 ± 0.07	<0.001
Relative fat mass	25.3 ± 0.07	39.4 ± 0.09	<0.001
Smoking (%)	1050 (74.2)	62 (5.7)	<0.001
Drinking (%)	432 (30.5)	53 (4.8)	<0.001
Aerobic physical activity (%)	578 (45.2)	276 (29.7)	<0.001
Resistance exercise (%)	306 (21.6)	68 (6.2)	<0.001
Household income (%)			<0.001
Quartile 1 (lowest)	562 (40.1)	567 (52.2)	
Quartile 2	435 (31.0)	288 (26.50	
Quartile 3	231 (16.5)	136 (12.5)	
Quartile 4 (highest)	174 (12.4)	96 (8.8)	
Education level (%)			<0.001
≤Elementary school	508 (39.8)	703 (74.9)	
Middle school	229 (17.9)	105 (11.2)	
High school	329 (25.8)	84 (9.0)	
≥University	210 (16.5)	46 (4.9)	
Basal metabolic rate (kJ/day)	6173.8 ± 8.6	4875 ± 10.0	<0.001
Handgrip strength (kg)	33.6 ± 0.1	20.2 ± 0.1	<0.001
Comorbidity number	1.3 ± 0.02	1.3 ± 0.03	0.452

Abbreviation: BMI, body mass index. Values are presented as mean ± standard deviation or number (percentage). * *p*-Values were assessed by weighted analysis of variance or weighted chi-square test.

**Table 2:** Demographic and clinical characteristics according to handgrip strength quartiles (kg).

Men Handgrip Strength (kg)						Women Handgrip Strength (kg)				
Variable	Q1 (~29.1)	Q2 (29.2–33.9)	Q3 (34.0–38.1)	Q4 (38.2–59.4)	*p*-Value for Trend *	Q1 (~16.8)	Q2 (16.9–20.5)	Q3 (20.6–23.8)	Q4 (23.9–37.1)	*p*-Value for Trend *
Unweighted N	356	348	358	354		272	276	276	272	
Age (years)	75.3 ± 0.2	73.2 ± 0.2	71.0 ± 0.2	69.4 ± 0.1	<0.001	76.7 ± 0.2	73.9 ± 0.2	72.4 ± 0.2	7.07 ± 0.2	<0.001
BMI (kg/m^2^)	22.8 ± 0.1	23.2 ± 0.09	23.7 ± 0.1	24.7 ± 0.1	<0.001	23.4 ± 0.2	24.1 ± 0.2	24.2 ± 0.1	24.6 ± 0.1	<0.001
Relative fat mass	25.0 ± 0.1	25.0 ± 0.1	25.4 ± 0.1	25.7 ± 0.1	<0.001	39.6 ± 0.2	39.9 ± 0.2	39.1 ± 0.1	39.1 ± 0.1	0.007
Smoking (%)	253 (71.0)	255 (72.4)	269 (72.4)	273 (77.6)	0.022	21 (10.0)	18 (6.5)	10 (5.1)	13 (4.6)	<0.001
Drinking (%)	93 (24.4)	109 (30.60	114 (34.0)	116 (33.0)	<0.001	8 (3.8)	10 (3.6)	15 (6.3)	20 (7.0)	<0.001
Aerobic physical activity (%)	105 (34.4)	131 (44.1)	165 (49.5)	177 (52.7)	<0.001	38 (13.2)	68 (27.0)	83 (31.0)	87 (36.3)	<0.001
Resistance exercise (%)	40 (10.4)	52 (16.5)	100 (27.6)	114 (33.0)	<0.001	4 (1.2)	14 (4.8)	27 (11.0)	23 (7.6)	<0.001
Household income (%)					<0.001					<0.001
Quartile 1 (lowest)	197 (54.8)	149 (43.1)	124 (34.5)	92 (25.8)		89 (35.6)	68 (27.0)	68 (23.8)	52 (20.9)	
Quartile 2	88 (24.5)	109 (26.0)	116 (25.8)	122 (28.7)		71 (25.0)	72 (24.5)	66 (23.0)	72 (26.5)	
Quartile 3	47 (13.7)	47 (13.0)	60 (18.3)	77 (23.6)		49 (15.7)	62 (21.4)	69 (25.0)	74 (27.5)	
Quartile 4 (highest)	22 (7.0)	36 (10.8)	56 (17.3)	60 (15.3)		61 (23.8)	70 (27.2)	73 (28.3)	71 (26.3)	
Education level (%)					<0.001					<0.001
≤Elementary school	170 (55.5)	140 (47.8)	108 (30.7)	90 (28.3)		182 (90.6)	189 (78.4)	170 (66.8)	162 (68.3)	
Middle school	48 (16.7)	56 (16.8)	54 (15.7)	71 (20.1)		13 (4.8)	20 (8.1)	29 (12.3)	43 (17.4)	
High school	59 (19.1)	62 (17.7)	106 (33.9)	102 (32.8)		8 (3.5)	15 (6.0)	35 (13.2)	26 (9.9)	
≥University	24 (8.7)	56 (17.7)	62 (19.7)	68 (18.8)		2 (1.1)	16 (7.4)	18 (7.7)	10 (4.4)	
Comorbidity number	1.2 ± 0.05	1.4 ± 0.05	1.3 ± 0.05	1.2 ± 0.04	0.091	1.2 ± 0.07	1.4 ± 0.06	1.4 ± 0.05	1.2 ± 0.02	0.001

Abbreviation: BMI, body mass index. Values are presented as mean ± standard deviation or number (percentage); * *p*-values were assessed by weighted analysis of variance or weighted chi-square test.

**Table 3:** Univariate and multivariate linear regression analysis of basal metabolic rate and studied variables in men.

Variable	Univariate		Multivariate (Enter)		Multivariate (Stepwise)	
	BMR		BMR		BMR	
	*r* or Mean ± SD	*p*-Value	B (SE)	*p*-Value	B (SE)	*p*-Value
Age	−0.255	<0.001	−1.6 (1.4)			
BMI	0.883	<0.001	166.0 (4.0)	<0.001	165.8 (4.0)	<0.001
RFM	0.679	<0.001	−21.6 (3.0)	<0.001	−21.6 (2.9)	<0.001
HGS	0.396	<0.001	10.9 (1.0)	<0.001	11.5 (0.9)	<0.001
Smoking		0.353				
No	6152.9 ± 492.4		Ref (0)			
Yes	6181.0 ± 498.4		25.5 (14.2)	0.078	28.1 (14.1)	0.047
Drinking		0.959				
No	6174.2 ± 504.7		Ref (0)			
Yes	6172.7 ± 479.0		12.9 (12.8)	0.309		
Aerobic physical activity		0.124				
No	6167.2 ± 506.8		Ref (0)			
Yes	6209.9 ± 475.9		−17.4 (12.0)	0.182		
Resistance exercise		<0.001				
No	6138.7 ± 495.4		Ref (0)			
Yes	6300.8 ± 481.8		−12.4 (14.2)	0.382		
Household income		<0.001				
Quartile 1 (lowest)	6099.9 ± 499.7		Ref (0)			
Quartile 2	6156.6 ± 490.1		−37.1 (14.6)	0.011	−34.9 (12.6)	0.006
Quartile 3	6297.6 ± 474.0		−7.4 (17.9)	0.681		
Quartile 4 (highest)	6298.4 ± 488.2		13.6 (20.7)	0.509		
Education level		<0.001				
≤Elementary school	6103.1 ± 492.2		Ref (0)			
Middle school	6149.6 ± 503.6		29.4 (16.9)	0.082		
High school	6241.5 ± 479.1		51.3 (15.4)	0.001	39.4 (13.9)	0.005
≥University	6325.5 ± 472.1		87.2 (19.1)	<0.001	75.1 (16.6)	<0.001
Comorbidity number	0.125	<0.001	10.1 (4.9)	0.039	10.3 (4.9)	0.035

Abbreviation: BMI, body mass index; RFM, relative fat mass; HGS, handgrip strength. Data are presented as mean ± standard deviation (SD) or number (percentage) or B (standard error (SE)). *p* is calculated via univariate and multivariate (enter, stepwise) linear regression.

**Table 4:** Univariate and multivariate linear regression analysis of basal metabolic rate and studied variables in women.

Variable	Univariate		Multivariate (Enter)		Multivariate (Stepwise)	
	BMR		BMR		BMR	
	*r* or Mean ± SD	*p*-Value	B (SE)	*p*-Value	B (SE)	*p*-Value
Age	−0.226	<0.001	−7.0 (1.5)	<0.001	−8.0 (1.4)	<0.001
BMI	0.867	<0.001	128.6 (3.3)	<0.001	129.4 (3.3)	<0.001
RFM	0.594	<0.001	−14.5 (2.7)	<0.001	−15.7 (2.7)	<0.001
HGS	0.333	<0.001	11.8 (1.4)	<0.001	12.2 (1.4)	<0.001
Smoking		0.449				
No	4878.0 ± 451.8		Ref (0)			
Yes	4832.9 ± 520.0		−11.7 (29.3)	0.689		
Drinking		0.501				
No	4873.4 ± 452.0		Ref (0)			
Yes	4916.5 ± 527.7		−3.3 (27.8)	0.905		
Aerobic physical activity		0.739				
No	4884.2 ± 458.7		Ref (0)			
Yes	4873.5 ± 414.7		0.6 (14.3)	0.969		
Resistance exercise		0.137				
No	4870.2 ± 459.5		Ref (0)			
Yes	4955.0 ± 389.2		18.1 (25.0)	0.47		
Household income		0.088				
Quartile 1 (lowest)	4849.3 ± 469.6		Ref (0)			
Quartile 2	4878.1 ± 438.9		14.0 (15.5)	0.369		
Quartile 3	4929.6 ± 416.9		33.3 (20.6)	0.106		
Quartile 4 (highest)	4952.9 ± 477.8		27.6 (23.3)	0.236		
Education level		0.158				
≤Elementary school	4860.0 ± 452.2		Ref (0)			
Middle school	4952.2 ± 398.9		33.7 (21.0)	0.109		
High school	4893.9 ± 423.1		22.6 (23.8)	0.344		
≥University	4943.9 ± 440.9		94.2 (30.6)	0.002	94.9 (29.6)	0.001
Comorbidity number			17.2 (5.5)	0.002	18.3 (5.4)	0.001

Abbreviation: BMI, body mass index; RFM, relative fat mass; HGS, handgrip strength. Data are presented as mean ± standard deviation or number (percentage) or B (standard error). *p* is calculated via univariate and multivariate (enter, stepwise) linear regression.

**Table 5:** Trend analysis of basal metabolic rate and handgrip strength quartile (kg).

**Model**	**Male Handgrip Quartile (kg)**				
	**Q1 (~29.1)**	**Q2 (29.2~33.9)**	**Q3 (34.0~38.1)**	**Q4 (38.2~59.4)**	***p*-Value for Trend**
Unadjusted	5947.5 ± 13.1	6090.7 ± 12.5	6220.5 ± 16.1	6443.5 ± 16.9	<0.001
Model 1 *	6077.9 ± 7.9	6152.8 ± 6.6	6199.4 ± 7.1	6280.0 ± 8.4	<0.001
Model 2 ^†^	6127.2 ± 13.4	6190.0 ± 12.3	6233.1 ± 12.9	6309.2 ± 13.8	<0.001
**Model**	**Female Handgrip Quartile (kg)**				
	**Q1 (~16.8)**	**Q2 (16.9~20.5)**	**Q3 (20.6~23.8)**	**Q4 (23.9~37.1)**	***p*-Value for Trend**
Unadjusted	4683.0 ± 20.3	4810.8 ± 21.2	4924.9 ± 18.5	5072.0 ± 12.1	<0.001
Model 1 *	4797.2 ± 8.2	4811.2 ± 10.8	4895.3 ± 8.7	4975.5 ± 6.7	<0.001
Model 2 ^†^	4859.9 ± 15.4	4848.3 ± 17.8	4916.4 ± 13.0	5005.1 ± 14.7	<0.001

Values are presented as mean ± standard deviation assessed by ANCOVA test.; * Model 1: adjusted for age and BMI; ^†^ Model 2: adjusted for age, BMI, relative fat mass, resistance exercise, aerobic physical activity, comorbidity number, household income, education level, smoking, and alcohol use.
